# Spatial and Temporal Variations in Arsenic Exposure via Drinking-water in Northern Argentina

**Published:** 2006-09

**Authors:** Gabriela Concha, Barbro Nermell, Marie Vahter

**Affiliations:** ^1^ Swedish National Food Administration, Toxicology Division, 75126, Uppsala; ^2^ Institute of Environmental Medicine, Karolinska Institutet, 171 77 Stockholm, Sweden

**Keywords:** Arsenic, Exposure, Urine, Hair, Inter-individual variation, Women, Drinking-water, Food, Argentina

## Abstract

This study evaluated the spatial, temporal and inter-individual variations in exposure to arsenic via drinking-water in Northern Argentina, based on measurements of arsenic in water, urine, and hair. Arsenic concentrations in drinking-water varied markedly among locations, from <1 to about 200 μg/L. Over a 10-year period, water from the same source in San Antonio de los Cobres fluctuated within 140 and 220 μg/L, with no trend of decreasing concentration. Arsenic concentrations in women's urine (3–900 μg/L, specific weight 1.018 g/mL) highly correlated with concentrations in water on a group level, but showed marked variations between individuals. Arsenic concentrations in hair (range 20–1,500 μg/kg) rather poorly correlated with urinary arsenic, possibly due to external contamination. Thus, arsenic concentration in urine seems to be a better marker of individual arsenic exposure than concentrations in drinking-water and hair.

## INTRODUCTION

Arsenic is ubiquitous in the environment and is usually present in small amounts (1.5–2 mg/kg) in the bedrock. However, the concentrations vary considerably, and leakage from naturally-occurring arsenic-rich minerals and sediments to ground water is a growing public-health problem worldwide. Millions of people in Asia, the American continent, and Europe are exposed to arsenic concentrations in drinking-water that far exceed the drinking-water quality guideline of 10 μg/L recommended by the World Health Organization (1, 2).

High levels of arsenic in ground water have previously been reported from several areas in Argentina, particularly in the Puna region in the Andes, the Chaco region, Córdoba, and the Pampean Plain (3–8). In la Francia, Córdoba, concentrations of arsenic as high as 3,000 μg/L in groundwater were recorded (3) and up to 600 μg/L in the Pampean Plain (4). It was reported that the number of locations with elevated arsenic concentrations of natural origin in water continued to grow. In addition, food may contain elevated arsenic concentrations (9–11).

The aim of the present study was to evaluate variations in human exposure to arsenic in the Puna region in the Andes in northwestern Argentina and the Chaco Salteño region in the north of Argentina, based on the studies we have carried out in the area over a 10-year period (9, 12) and on recent follow-up studies. We assessed arsenic exposure based on arsenic concentrations in drinking-water, urine, and hair to obtain as much information as possible on the exposure situation and dose. Urine is the major route of excretion of absorbed arsenic, and the concentration of arsenic in urine is commonly used for the evaluation of current exposure to arsenic on an individual level. In the case of continuous arsenic exposure via drinking-water, urinary excretion of arsenic is remarkably constant over time (13, 14). Inorganic arsenic binds to sulphydryl groups in the body, e.g. keratin in hair. Therefore, arsenic in hair is a useful biomarker of more long-term arsenic exposure (15).

## MATERIALS AND METHODS

### Study sites and subjects

The Puna is an arid highland surrounded by a mountain belt in the central part of the Andes, in the province of Salta in northern Argentina. The temperature varies from about -26 °C in July to about +30 °C (daytime) in December. The volcanic bedrock has a high content of arsenic associated with pyrite minerals (16). The main part of the study was conducted in San Antonio de los Cobres, with about 5,000 inhabitants, mainly indigenous, at an altitude of 3,800 m. The local economy is based on breeding of llamas, goats, and sheep. The staple diet of the population is mainly of animal origin (meat, milk), supplemented with vegetables, maize, and rice. The source of drinking-water in San Antonio de los Cobres is a natural spring, Agua de Castilla, located about one km outside the village. Arsenic exposure was also studied in the small Puna communities—Olacapato, Santa Rosa de los Pastos Grandes, and Tolar Grande (each with less than 200 inhabitants), located 60–187 km southwest and west of San Antonio de los Cobres.

The Chaco Salteño region is a semi-arid area located in the southeast of the province of Salta. Taco Pozo has 8,500 inhabitants; Joaquín V. Gonzalez, a village located only a few km from Taco Pozo, has about 13,400 inha-bitants, and Anta has about 400 inhabitants. The climate is much warmer than in the Andes, particularly in the winter. The most important industries are related to the production of cotton, tobacco, and wood, and the breeding of cattle, goats, and pigs, combined with the growing of maize, pumpkins, and squash. Most people use well-water either from private wells or from the public distribution system. For comparison, we also determined arsenic exposure in Salta, the capital of the province (at an altitude of about 1,000 m) and the nearby village Rosario de Lerma.

The present study focused on arsenic exposure in women who spend more time close to home than men, who were often far away for a long time for work. In San Antonio de los Cobres, women were recruited via the local radio and the hospital registers with the help of physicians and community health workers at the hospital. In the other villages, women were recruited with the assistance of personnel at the local health centres. Although it was not possible, for practical reasons, to select the women at random, measures were taken to get as wide a distribution of households as possible. Information on sources of drinking-water, water consumption, dietary habits, and time of residence in the area was obtained through personal interviews.

### Sample collection

Arsenic exposure was assessed by measuring arsenic in water, urine, and hair. Drinking-water was collected from the public water-distribution systems or private wells in 60-mL acid-washed polyethylene bottles after flushing the water for about one minute. To decrease the pH, an aliquot of 100 μL of concentrated nitric acid (p.a. Merck, Germany) was added. Spot-urine samples were collected in acid-washed plastic containers and were transferred immediately to 60 mL acid-washed polyethylene bottles. All the samples were frozen and kept at -20 °C until they were transported packed with cooling blocks to Sweden, where they were analyzed within a couple of months. We also collected hair samples from some women in the Puna region and Rosario de Lerma. A couple of mm thick bundle of hair in the back of the head was tied with a cotton thread and cut close to the scalp. The hair samples were washed with 1% Triton X—100 for about one hour, rinsed five times in de-ionized water, and dried overnight at room temperature prior to analysis. For comparison, we analyzed arsenic in hair previously collected from children, aged 3–15 years, from the same villages as the women, but with a lower degree of arsenic methylation (12).

### Analysis

Urinary concentration of inorganic arsenic and its metabolites (methylarsonic acid [MMA] and dimethylarsinic acid [DMA]), a commonly-used measure of individual exposure to inorganic arsenic (17), was determined by direct hydride generation-atomic absorption spectrophotometry (HG-AAS) after addition of HCl to 0.6 M (18, 19). Standard curves were prepared from solutions containing 70% DMA (Merck) and 30% As_2_O_5_ (Merck) in 0.6 M HCl (0–200 ng As), the average distribution in urine, as the peak areas differ for the different metabolites. Concentrations of total arsenic in water, urine, and hair were determined using HG-AAS after dry ashing of samples mixed with ashing aid solution (19, 20). Standard curves were prepared from As_2_O_5_ (Merck) solutions in 3 M HCl (0–200 ng As).

To compensate for variations in the dilution of urine, arsenic concentrations in urine were adjusted to the mean specific gravity (1.018 g/mL) measured by a hand refractometer (Atago, Japan). We preferred adjustment by specific gravity to that by creatinine, the most commonly-used adjustment method because the excretion of creatinine varies with meat intake and muscle mass, age, and physical activity (21). Also, adjusting for density was a practical approach in the field.

**Fig. 1.**
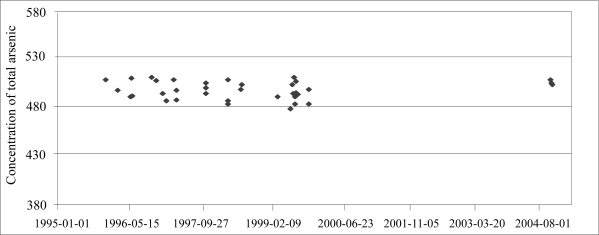
Obtained concentrations of sum of arsenic metabolites in NIST HL reference urine over the whole study period. Certified concentration of total arsenic is 480±100 μg/L. Reported concentrations of arsenic metabolites are 499 μg/L (22) and 481 μg/L (23)

To ascertain analytical accuracy, commercially-available reference materials, with certified or recommended arsenic concentrations, were included in each analysis. There was good agreement between obtained arsenic concentrations in the reference materials used and the reference values (Table 1), indicating good analytical quality. The arsenic reference values for NIST 2670 urine are for total arsenic only, while we measured the sum of arsenic metabolites. Other research groups have speciated arsenic in NIST HL urine and found that it contains mainly AsV (about 87%), the rest being DMA, MMA, and arsenobetaine, a common organic arsenic compound in seafood (22, 23). NIST LL urine contained about 80% DMA. To verify the arsenic performance over time, we plotted the results of reference urine over time (Fig. 1).

**Table 1.** Reference materials analyzed for arsenic together with collected samples in the present work. For arsenic in urine, both sum of arsenic metabolites and total arsenic are given, as the certified or recommended values are for total arsenic only

Reference material (certified or recommended values)	Matrix	Obtained arsenic concentrations and time of analyses
NIST 2670, high level (total As 480±100 μg/L)	Urine	Total As: 522±44 μg/L, n=119 (1997–1999)
As metabolites 499/481 μg/L*		As metabolites: 494±9, n=33 (1997–2004)
NIST 2670, low level (total As 60 μg/L)	Urine	Total As: 62±3.0 μg/L, n=17 (1997–1999)
As metabolites 60/44 μg/L*		As metabolites: 60±4, n=10 (1997–1999)
NIST 1643a, (76±7 μg/L)	Water	78±5.5 μg/L, n=16 (1994)
NIST 1643c, (82±1.2 μg/L)	Water	86±5.5 μg/L, n=20 (1995–1999)
NIST 1643d, (56.02±0.73 μg/L)	Water	56±0.3 μg/L, n=6 (2004)
GBW 09101 (590±70 μg/kg)	Hair	588±22 μg/kg, n=6 (1999–2001)

*Values reported by Le *et al*. and Mandal *et al*. (22, 23)

As=Arsenic

### Statistical analysis

Descriptive analysis of data included calculations of central tendency (mean/median) and variation (percentiles). Arsenic concentrations in urine were not normally distributed. Thus, both Spearman and Pearson correlation analyses were performed to evaluate associations between variables.

### Ethics

Because of the time period between urine collection and arsenic analysis, we could not inform the women about the arsenic concentrations in water and urine. However, all results were reported to the responsible physicians at the local hospitals and to the Ministry of Health in Salta, Argentina. Ethical approval for the studies was obtained from the Ministry of Health in Salta and from the ethical committee at Karolinska Institute, Stockholm, Sweden. When asked for written consent, the volunteers were informed about all parts of the study and the right to interrupt their participation at any time.

## RESULTS

Arsenic concentrations in drinking-water varied considerably among the different locations in both Puna and Chaco Salteño regions (Table 2). The concentrations of arsenic in drinking-water in San Antonio de los Cobres was around 200 μg/L, while it varied between 2.5 and 31 μg/L in the three small Puna communities. Arsenic concentrations in water in the Chaco Salteño region ranged from 6 to 250 μg/L, while the concentrations in Salta and the nearby village Rosario de Lerma were below 1 μg/L.

**Table 2.** Concentrations of total arsenic in drinking-water (groundwater) and surface water collected in different regions in the north of Argentina

Village	Water source	Arsenic concentration (μg/L)
Puna region		
San Antonio de los Cobres	Piped well-water	189*
Tolar Grande	Piped well-water	2.5
Olacapato	Piped well-water	14
St Rosa de los Pastos Grandes	Piped well-water	31
Chaco Salteño region		
Taco Pozo	Private well/piped well-water (n=2)	214, 216
Joaquín V. Gonzalez	Piped well-water (n=2)	5.5, 7.2
Anta	Private wells (n=6)	187 (110–250)‡
Salta		
Town of Salta	Public water distribution (groundwater)	0.5
Rosario de Lerma	Public water distribution (groundwater) (n=3)	0.6, 07, 0.6

*Average of all samples 1994–2004 (Table 3);

‡Mean and (range)

As=Arsenic

Over the entire 10-year study period in San Antonio de los Cobres, arsenic concentrations in the water distribution system fluctuated between 140 and 220 μg/L (Table 3). On some occasions, the concentrations varied among different sampling sites on the water distribution system (CV 4–27%). The concentration in the river running through the village was 780 μg/L in November 1994, 800 μg/L in September 1995, 820 μg/L in August 1996, and 997 μg/L in November 2004.

**Table 3.** Concentrations of total arsenic in drinking-water in San Antonio de los Cobres during 1994–2004. Water samples were collected from taps in private homes and from the water source, the natural spring Agua de Castilla

Sampling time	No. of samples	Arsenic concentration (μg/L)
April 1994	4	205±15 (192–217)*
November 1994	2	160, 157
	1	179**
September 1995	2	214, 216
May 1996	1	188
August 1996	3	162, 200, 218
October 1996	1	183
November 1997	5	143±38 (107–195)*
March 1999	3	140, 180, 187
	1	214**
November 2004	8	219±7.7 (207–229)*
Mean	31	186±26

*Mean, SD, and (range);

**Agua de Castilla

As=Arsenic

Personal interviews with participating women revealed that the length of residence in different villages ranged from six months to 76 years (average 27 years) and that the predominant source of drinking-water was local water. These women did not consume seafood, which may cause elevated concentrations of DMA in the urine (24) and, thus, be interpreted as exposure to inorganic arsenic. Arsenic concentrations in urine in San Antonio de los Cobres (overall average 289 μg/L) and Taco Pozo (366 μg/L) were about 30 times higher than those in the villages with low concentrations of arsenic (around 10 μg/L) in water (Table 4). Arsenic concentrations in urine in Anta were only about 40% of those in San Antonio and Taco Pozo, despite similar concentrations of arsenic in water.

**Table 4.** Concentrations of arsenic metabolites in urine (μg/L, adjusted to specific gravity of 1.018 g/mL) of women from the Puna and Chaco regions in Northern Argentina. Data represent median values (range)

Village	Arsenic metabolites in urine (μg/L)
Puna region	
San Antonio de los Cobres	
n=11 (April 1994)	288 (121–456)
n=15 (November 1997)	238 (98–325)
n=7 (March 1999)	232 (138–254)
n=96 (November 2004)	301 (54–899)
Tolar Grande, n=5	15 (10–16)
Olacapato, n=5	26 (11–44)
Santa Rosa de los Pastos	
Grandes, n=5	55 (36–64)
Chaco Salteño region	
Taco Pozo, n=12	366 (85–574)
Joaquín V. Gonzalez, n=5	10 (6.0–18)
Anta, n=10	141 (72–300)
Salta	
Rosario de Lerma, n=12	7.2 (3.0–21)

**Fig. 2.**
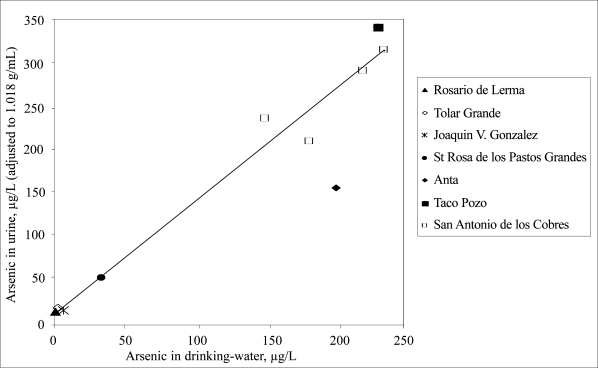
Association between average concentrations of arsenic metabolites in urine in eight villages in Northern Argentina and corresponding concentrations of arsenic in drinking-water

**Fig. 3.**
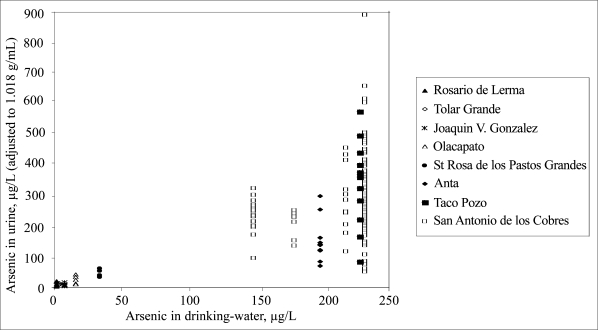
Individual variation in concentrations of arsenic metabolites in urine in relation to arsenic concentrations in drinking-water

There was a significant correlation (r=0.96, p<0.001) on a group basis between the concentrations of arsenic in urine and drinking-water (Fig. 2), but a marked variation in urinary arsenic among individuals in each village (Fig. 3).

**Fig. 4.**
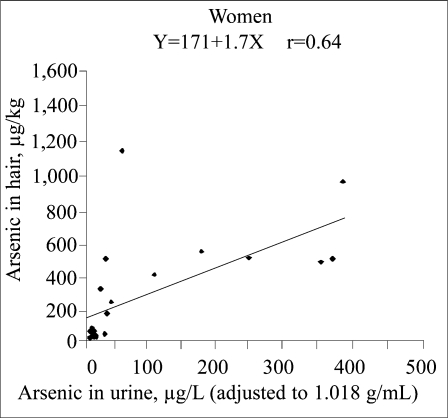
Correlation between concentrations of arsenic in hair and urine from women in San Antonio de los Cobres, small Puna villages, and Rosario de Lerma

**Fig. 5.**
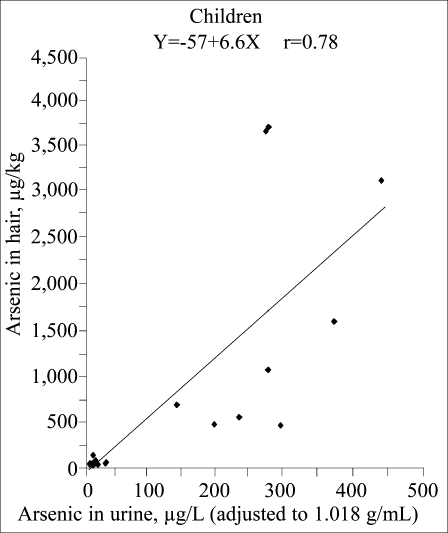
Correlation between concentrations of arsenic in hair and urine from children in San Antonio de los Cobres and Rosario de Lerma

Arsenic concentrations in the hair of women ranged from 24 to 72 μg/kg (median 33 μg/kg, n=9) in Rosario de Lerma, from 42 to 1,500 μg/kg (median 160 μg/kg, n=10) in the small Puna villages, and from 515 to 1,241 μg/kg (median 651 μg/kg, n=8) in San Antonio de los Cobres. The concentrations in hair of children were 24–149 μg/kg (median 50 μg/kg, n=15) in Rosario de Lerma and 521–4,250 μg/kg (median 1,746 μg/kg, n=12) in San Antonio de los Cobres. There was a statistically significant association between concentrations of arsenic in hair and urine for both women (r=0.64, p=0.001) and children (r=0.78, p<0.0001), but with steeper slope for the children (Fig. 4–5). However, as shown in Figures 4 and 5, there was a noticeable variation in arsenic concentrations in hair for similar concentrations in urine.

## DISCUSSION

The results of the present study showed that there was a marked variation in arsenic exposure via drinking-water in the Puna and Chaco Salteño regions in Northern Argentina, which is in agreement with previous reports on variations in arsenic concentrations in groundwater (7). In some areas, arsenic concentrations were 20 times higher than the WHO's drinking-water guideline of 10 μg/L (2) and four times higher than the local maximum permissible level of 50 μg/L (www.anmat.gov.ar). The highest concentrations were found in San Antonio de los Cobres, Taco Pozo, and Anta, with a total population of about 14,000. Arsenic concentrations in Salta, the capital of the province, and in the nearby village Rosario de Lerma were well below the standards.

In San Antonio de los Cobres, where water from the spring Agua de Castilla is distributed throughout the village, the observed variations in arsenic concentrations in water among the sampling sites at some occasions may indicate co-precipitation of arsenate (AsV) with iron (25) in water pipes, particularly in parts of the distribution system with low water turnover. The measurement error (CV of about 5% in water analyses, Table 1) can explain only a small part of the variation across households (CV up to 27 %). The assay used did not distinguish between different oxidation states of inorganic arsenic, but, as the water was chlorinated before entering the village distribution system, it is likely that most arsenic in the water was in the form of arsenate (AsV). The concentration of iron was about 0.8 mg/L, which implies risk of precipitation (25). Since arsenic and iron often occur concurrently in groundwater (26), our results demonstrate that the arsenic concentration in ingested water may differ markedly from that in the water source, particularly in cases where the water is kept for a longer period in the tubing or other containers before consumption.

Repeated sampling of drinking-water in San Antonio de los Cobres at nine different occasions between 1994 and 2004 showed no trend of decreasing arsenic concentration. Thus, the implemented mitigation activities in the form of a water-treatment plant have not reduced the arsenic concentration in water. There was a fairly constant fluctuation (140–220 μg/L) over time, which may be related to variations in precipitation, but also to variation in rainfall (7). According to the Argentinean National Meteorological Service (www.meteofa.mil.ar), the average annual precipitation in the Puna region is only about 100 mm, with less than 5 mm during the winter season, although it may vary greatly from year to year. Also, arsenic concentration in the river, which was about four times higher than that in well-water, was fairly constant during the 10-year study period. Although river water is not used as drinking-water for inhabitants, it illustrates the fairly constant arsenic concentration in the waters in the area. There are very few previous reports on temporal variation of arsenic in well-water, but those also indicate fairly stable arsenic concentrations over several years (26–28). As the situation may vary depending on the hydrogeological conditions, more studies are needed to understand how arsenic concentrations in groundwater fluctuate over time and which factors, if any, are important for significant changes in the concentration.

On a group basis, there was a strong correlation between the concentrations of arsenic in urine and drinking-water, demonstrating the suitability of urine as biomarker of arsenic exposure. A low consumption of beverages other than local drinking-water in investigated groups (9) is likely to have contributed to the strong correlation. The high intake of local arsenic-rich water also resulted in high arsenic concentrations in urine compared to water. The ratio between urinary and water arsenic concentrations was 1.5:1, while previous studies in population groups in the USA and Taiwan showed ratios of 1:1 or lower (14, 29–31). To what extent the differences in metabolism of arsenic may contribute to the differences in urine to water ratios are not yet elucidated. There is evidence that the arsenic methylation efficiency may influence the retention of arsenic in the body, in particular that a higher fraction of DMA and a lower fraction of MMA in the urine are associated with excretion of a higher fraction of the ingested dose of arsenic (17). Thus, the fact that the indigenous people in the study areas in Northern Argentina excrete less MMA in urine than all other people studied (9, 12), may have contributed to the high concentrations of arsenic in urine compared to water. It can be speculated that this also explains the lower urinary arsenic concentrations in women in Anta compared to those in San Antonio de los Cobres and Taco Pozo, despite similar concentrations in drinking-water. The Anta women had more than three times higher percentage of MMA in urine (median 7.4%, range 3.2–18%) than had women in the other villages (2.2%) (12). This variation in MMA excretion may be related to ethnicity, with indigenous people producing less MMA than those of Spanish or mixed origin (9, 30). Our ongoing studies on genetic polymorphisms will hopefully increase the understanding of factors influencing the metabolism of arsenic.

Although many investigated individuals used the same public drinking-water, there was a marked inter-individual variation in arsenic concentration in urine. In the 2004 sampling in San Antonio de los Cobres, urinary arsenic varied between 54 and 900 μg/L for women with drinking-water from the same source. This cannot be explained by the use of spot-urine samples for the analysis of arsenic concentrations, as there was no major variation in urinary arsenic concentrations over several days (13). Most women had lived in the respective area for long time and, most likely, the arsenic, with a whole-body half-time of a few days only, had reached steady state in their bodies. Probably, the main reason for the inter-individual variation in urinary arsenic was variation in water consumption, which is known to vary greatly among individuals (32). The reported daily intake of drinking-water and other water-based fluids ranged from 0.2 to 1.2 litres. Although the exact amount of fluid intake is difficult to assess based on recall data (33), the reported water consumptions indicate a considerable variation.

A variation in the intake of arsenic via food may also contribute to the variation in urinary arsenic relative to arsenic concentration in water. High concentrations of arsenic, about 400 μg/kg, were found in soup and polenta in San Antonio de los Cobres and Taco Pozo (9, 12). Probably, most arsenic in food derived from the water used for cooking. Polenta sampled in Tolar Grande, one of the small Puna villages with low concentrations of arsenic in drinking-water, contained only about 10 μg/kg, indicating low concentrations in the maize used for the polenta.

There was a rather poor correlation between arsenic in hair and urine. Probably, this was mainly due to contamination of hair from water during hair washing and from dust (15). Among the women, the highest arsenic concentration in hair—1,500 μg/kg—was from a woman with only 64 μg/L in the urine. We do not know the reason for this, but it might be due to hair washing in arsenic-rich water, e.g. from hot springs. In the old Pompeya spa, about 10 km north of San Antonio de los Cobres, water contained as much as 6,000 μg/L (9). The thermal water in this and other spas is still used for bathing, but not as drinking-water. Unless water is ingested during bathing, the exposure is low, as the percutaneous absorption of arsenic is negligible compared to intestinal absorption (34). However, the arsenic will bind to the external sulphydryl groups in the hair. Two investigators tested arsenic concentrations in their hair after visiting San Antonio de los Cobres. After a few days of visit with no hair washing, the concentrations in hair were 33 and 78 μg/kg (range 12–73 and 26–176 μg/kg) for the two investigators respectively, i.e. similar to that in people without particular arsenic exposure (usually less than 200 μg/kg). After washing hair once with local water (about 200 μg/L), the concentrations in hair were 395 and 461 μg/kg, with small variations along the length of the hair strands (range for 1-cm sub-samples: 242–556 and 376–589 μg/kg respectively). However, the concentrations in urine were similar at the two occasions (48/35 and 55/40 μg/L). This clearly supports external binding of arsenic on hair, which is not possible to remove with the washing procedure for hair samples before analysis.

The variation in arsenic concentrations in hair compared to urine may also be due to variation in arsenic metabolism. The higher concentrations in hair of children compared to women from San Antonio de los Cobres, despite similar concentrations in the urine, may be related to the low arsenic-methylating capacity in the children. The children had almost 50% of inorganic arsenic in their urine compared to 25% in women (12), and it is mainly inorganic arsenic (>90%) that is incorporated into hair (23, 35). Also, children may be more exposed to dust during playing.

It can be concluded that arsenic concentrations in drinking-water in Northern Argentina vary markedly between areas, but there is no indications of major temporal variations. There are certain fluctuations in arsenic concentrations over time and within a distribution system. The major variations in the concentrations of arsenic in urine indicate variation in the intake of arsenic via water and food, possibly also variations in the metabolism of arsenic. Arsenic concentrations in drinking-water and hair do not provide reliable measures of individual arsenic exposure.
